# Fast, Highly-Sensitive, and Wide-Dynamic-Range Interdigitated Capacitor Glucose Biosensor Using Solvatochromic Dye-Containing Sensing Membrane

**DOI:** 10.3390/s16020265

**Published:** 2016-02-20

**Authors:** Md. Rajibur Rahaman Khan, Alireza Khalilian, Shin-Won Kang

**Affiliations:** School of Electronics Engineering, Kyungpook National University, 80 Daehakro, Bukgu, Daegu 41566, Korea; rajibur@ee.knu.ac.kr (Md.R.R.K.); alireza.khalilian991@gmail.com (A.K.)

**Keywords:** interdigitated capacitor, glucose biosensor, solvatochromic dye, dielectric constant, sensing element

## Abstract

In this paper, we proposed an interdigitated capacitor (IDC)-based glucose biosensor to measure different concentrations of glucose from 1 μM to 1 M. We studied four different types of solvatochromic dyes: Auramine O, Nile red, Rhodamine B, and Reichardt’s dye (R-dye). These dyes were individually incorporated into a polymer [polyvinyl chloride (PVC)] and N,N-Dimethylacetamide (DMAC) solution to make the respective dielectric/sensing materials. To the best of our knowledge, we report for the first time an IDC glucose biosensing system utilizing a solvatochromic-dye-containing sensing membrane. These four dielectric or sensing materials were individually placed into the interdigitated electrode (IDE) by spin coating to make four IDC glucose biosensing elements. The proposed IDC glucose biosensor has a high sensing ability over a wide dynamic range and its sensitivity was about 23.32 mV/decade. It also has fast response and recovery times of approximately 7 s and 5 s, respectively, excellent reproducibility with a standard deviation of approximately 0.023, highly stable sensing performance, and real-time monitoring capabilities. The proposed IDC glucose biosensor was compared with an IDC, potentiometric, FET, and fiber-optic glucose sensor with respect to response time, dynamic range width, sensitivity, and linearity. We observed that the designed IDC glucose biosensor offered excellent performance.

## 1. Introduction

The main purpose of a glucose sensor is to detect and monitor the level of glucose under several conditions. A glucose sensor has applications in various fields such as the health industry, agriculture, and the food and beverage industry. In the field of medical diagnosis/health industry, a glucose sensor is used to measure blood sugar levels. Diabetes mellitus, which is commonly known as diabetes, is a metabolic disease in which a person has high levels of glucose (sugar) in the blood. This high level of sugar in blood will cause hazardous complications, such as kidney failure, cardiovascular disease, stroke, damage to the blood vessels, foot ulcers, eye injuries, and degenerative nerve diseases [1,2]. According to the International Diabetes Federation, approximately 381 million people had diabetes in 2013 [3,4]. In the agricultural sector, fish farms use glucose sensors for checking the health of fish to rapidly detect abnormalities [5]. According to this research, the situation of respiratory or nutritional disturbance and the degree of stress in fish is closely correlated with the blood glucose levels. In addition, glucose sensors are under heavy demand in the food and beverage industries [6] for measuring the glucose in industrial products, such as sport drinks and fruit juices. 

Currently, there are many ongoing studies on the glucose biosensor, including colorimetry [7,8], conductometry [9], electrochemical methods [10], fluorescent spectroscopy [11], and optical methods [12,13,14,15,16,17]. Among of the above mentioned methods, electrochemical glucose sensors have enticed the most attention because of their sensitivity, low response time, high selectivity, high stability, low cost, and lower detection limit. Potentiometry [18,19,20,21], voltammetry [22], and amperometry [23,24,25,26,27,28,29] measurement techniques are the member of the electrochemical glucose biosensors.

In 2010, Ali *et al.* proposed a zinc-oxide-nanowire-based potentiometric glucose microsensor to detect low concentrations of glucose [19]. The advantages of this microsensor include its sensitivity, selectivity, low response time, and stability. The main disadvantage of this sensor is its low dynamic range of 0.5–1000 µM. Miaoa *et al.* proposed a highly sensitive amperometric glucose sensor having a low response time and good stability and reproducibility [26]. Although this glucose sensor had some advantages, it had a low dynamic range of approximately 40 mM. This sensor cannot detect glucose at less than 5 µM. Wang *et al.* proposed a strip based colorimetric optical glucose sensor [7]. This sensor is known for its easy fabrication, low cost, lack of requirement for a light source or auxiliary circuits, and its ability to detect various concentrations of glucose in real time. However, this sensor lacks a low dynamic range, and its response time is high (approximately 5 min). 

A sensor using an interdigitated microelectrode (IDμE) was proposed by Huang *et al.* to detect various concentrations of glucose [30]. The researchers did not use any sensing membranes or immobilizing enzymes on the microelectrode surface. The construction and principle of operation of this sensor are easy, but the sensor has several disadvantages including low dynamic range and lack of selectivity. A MOSFET-based electrochemical glucose sensor was developed by Ali *et al.* in 2009 [31]. In their research, on the Ag wire they grown the ZnO nanowires, and the enzyme glucose oxidase was immobilized on the ZnO nanowires to make a sensing electrode. Then this electrode was directly connected to the MOSFET gate. The sensor had a short response time and good stability, but the detection range was small (approximately 1–100 µM). 

In this study, we designed a highly sensitive, cheap, easy to prepare, wide-dynamic-range, and highly stable IDC glucose biosensing system whose operational method is based on the capacitor principle. We proposed multichannel lipid-containing IDC taste sensing system in [32]. This time we proposed solvatochromic-dye-containing IDC glucose biosensor. To the best of our knowledge, we report for the first time, an IDC glucose biosensing system using solvatochromic-dye-containing sensing membrane. To make/prepare the respective dielectric/sensing solution of the IDC sensing elements, we incorporate four kinds of solvatochromic dyes: Auramine O, Nile-red, Rhodamine B, and Reichardt’s dye (R-dye). These dyes were individually incorporated into a polyvinyl chloride (PVC) and N,N-Dimethylacetamide (DMAC) solution. These dielectric/sensing solutions were then used as sensing membranes and placed into the interdigitated electrodes (IDEs) by a spin coater to prepare four kinds of IDC sensing elements. 

When the IDC sensing element is immersed to the glucose solution, the dielectric constant of the sensing membrane of the IDC changes owing to the charge transfer character of the solvatochromic dye in the sensing membrane. Since, the dielectric constant of the sensing membrane change, which corresponds to change the capacitance of the IDC as well as the alter the amplitude across the IDC sensing element. The developed IDC sensing system can successfully measure low glucose concentrations. In our experiment, we also observed the sensing performance of sucrose, and we obtained a good response. The proposed IDC glucose biosensor was compared with a IDC, potentiometric, field effect transistor (FET), and fiber-optic glucose sensor with respect to response time, dynamic range width, sensitivity, and linearity. The designed IDC glucose biosensing system has better performance. 

## 2. Principle of Operation and Theory

**Figure 1:**
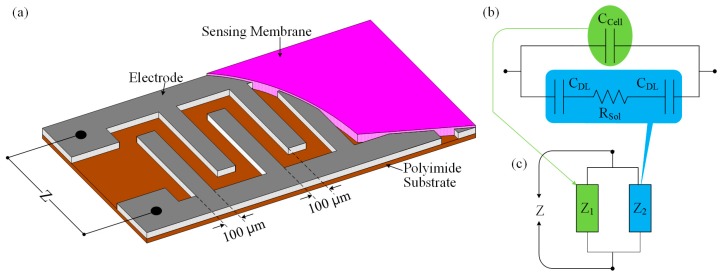
Interdigitated capacitor sensor: (**a**) schematic diagram of IDC sensing element; (**b**) simplified electrical circuit of the IDC; and (**c**) analogy of Figure 1b.

Figure 1a shows the schematic diagram of the IDC glucose biosensing element. The operation principle of the proposed interdigitated glucose biosensor is based on the electrodynamics of two parallel-plate capacitors. When the IDC sensing element is placed into the glucose solution, then its dielectric/sensing material (which was placed into the IDE) reacts with the glucose solution. As a result, the dielectric constant of the IDC changes, which also correspond to changes of the capacitive reactance of the sensing element. Therefore, the sensing response of the IDC can be obtained, by observing the change in the IDC sensing element’s voltage or the capacitive reactance of the IDC. Figure 1b shows the simplified equivalent electrical circuit of the dipped IDC into the glucose solution and the analogy of the electrical circuit in Figure 1b can be represented as in Figure 1c. 

At high frequencies, the impedance Z can be written by the mathematical equation as [32]:

(1) $$Z=\frac{R_{\mathrm{Sol}}}{1+2\pi fC_{\mathrm{Cell}}R_{\mathrm{Sol}}}$$

where $R_{\mathrm{Sol}}$ is the resistance of the medium solution and $C_{\mathrm{Cell}}$ is the cell capacitance, which in turn is connected in parallel with a series combination of $R_{\mathrm{Sol}}$ and two double-layered capacitors $C_{\mathrm{DL}}$. $R_{\mathrm{Sol}}$ and $C_{\mathrm{Cell}}$ can be written by the following equations [33]:

(2) $$R_{\mathrm{Sol}}=\frac{K_{\mathrm{Cell}}}{\sigma_{\text{Sol}}}$$

and

(3) $$C_{\mathrm{Cell}}=\frac{\varepsilon_{0}\varepsilon_{\text{R-Sol}}}{K_{\mathrm{Cell}}}$$

where $K_{\mathrm{Cell}}$ and $\sigma_{\text{Sol}}$ are the cell constant and electrolyte conductivity, respectively. $\varepsilon_{0}$ is the absolute and $\varepsilon_{\text{R-Sol}}$ is the relative dielectric constant of the medium.

If a constant current $I_{C}$ flows through the IDC sensing element, then the variation of the IDC-sensing-element’s voltage can be expressed by the following mathematical equation:

(4) $$\Delta V_{C}=I_{C}\Delta Z$$

## 3. Experimental Details

### 3.1. Preparation Process of the Interdigitated Electrode

In our experiment, we prepared the interdigitated electrode (IDE) of size 4 cm × 2 cm with 40 pairs of fingers on a polyimide (PI) substrate. We described the preparation process of the IDE in detail in [32]. In this experiment, the thickness, the width per finger, and the distance between the fingers of the IDE was approximately 54 μm, 103 μm and 103 μm, respectively, and was measured by a scanning electron microscope (SEM) (S-4800, Hitachi, Ibaraki, Japan). 

### 3.2. Preparation Process of the Sensing Solution and the Interdigitated Glucose Biosensing Element 

**Figure 2:**
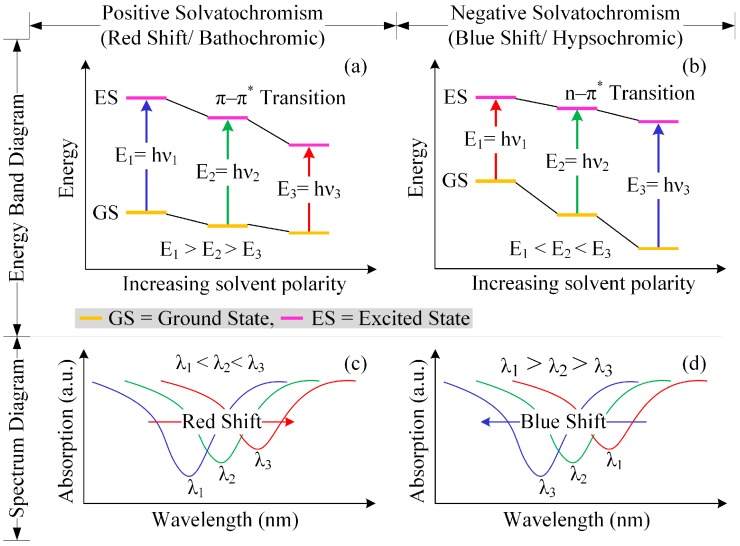
Representation of solvatochromism by: (**a**) energy band diagram of positive solvatochromism; (**b**) energy band diagram of negative Solvatochromism; (**c**) spectrum diagram of positive solvatochromism; and (**d**) spectrum diagram of neagative solvatochromism.

The ability of a chemical substance to change color owing to a change in solvent polarity is known as solvatochromism. Two types of solvatochromism are available: positive and negative solvatochoramism [34]. The chemical compound which has solvatochromic properties is called solvatochromic dye. In the case of positive solvatochromism, the excited state is more polar than the ground state and its stabilization is occurred at the excited state by increasing solvent polarity. Therefore, when the solvent become more polar, then the transition energy between the two electronic state is decrease, which correspond to reduce the energy band gap between the ground and the excited state and the light absorption peak is shift to higher wavelength (bathochromic shift) in the spectrum [35,36]. The opposite effect occurs in the case of negative solvatochromism. Solvatochromic dye has electrical as well as optical properties. The representation of solvatochromism by the energy band diagram as well as spectrum diagram are shown in Figure 2.

Four different kinds of solvatochromic dyes [Auramine O, Nile red, Rhodamine B, and Reichardt’s dye (R-dye)] and one polymer [polyvinyl chloride (PVC)] and N,N-Dimethylacetamide (DMAC) solution were used to fabricate the four different kinds of sensing/dielectric materials of the IDC biosensing elements. We bought all chemicals from the Sigma-Aldrich Chemical Corporation and utilized those chemicals without any purification. In our experiment, when the solvatochromic dye containing a sensing membrane comes into contact with a glucose solution, then the chemical properties of the sensing membrane change, which change the dielectric properties of the sensing membrane as well as alters the IDC-sensing-element’s capacitance. 

**Figure 3:**
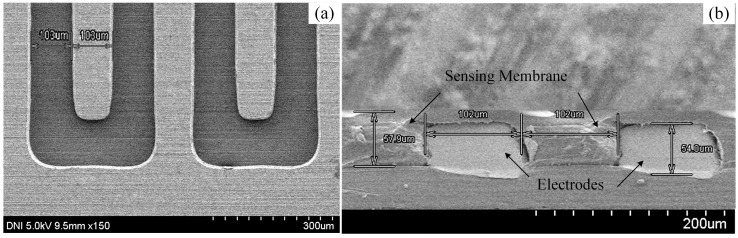
SEM images of the preparead IDE (**a**) Top view without sensing membrane; and (**b**) cross-sectional view of the IDE with sensing membrane.

To prepare the four types of sensing solutions for the proposed IDC biosensing element, we used the following procedure: First, 0.4 wt % of each solvatochromic dye was individually dissolved into 92.2 wt % of DMAC solution and sonicated for approximately 5 min to make solution-A. We mixed 7.4 wt % of PVC to solution-A and sonicated again for approximately 5 min to make four kinds of sensing solutions for the IDC. We washed the IDE with acetone, methanol, and DI water, and dried it with N_2_ gas. We took 0.5 mL of sensing solution and deposited it on the surface of the IDE. A spin-coater was used to insert the sensing solution properly into the IDE and to get a smooth surface over three stages of spinning for fabricating the IDC: 200 rpm for 5 s, 500 rpm for 5 s, and lastly, 1000 rpm for 20 s. Then, the IDC was dried overnight at the room temperature. The SEM images of the fabricated IDC sensing element are shown in Figure 3. 

The sensing membrane’s thickness of the developed IDC glucose biosensing element was approximately 57.9 µm and was measured by a SEM. Since the proposed IDC sensing element works on the principle of a capacitor, if the sensing membrane’s thickness of IDC reduces than the thickness of the IDE, then, the capacitance of the IDC reduces and gives the highest capacitance when the sensing membrane thickness is approximately equal to the thickness of the IDE. In our experiment we observed the capacitance of the Nile-red-containing sensing element at 1 mM of glucose concentration for different thickness of the sensing membrane such as: 45 µm, 57.9 µm, and 65 µm and we obtained the highest capacitance about 483 pF, at 57.9 µm of sensing membrane of IDC. Therefore, in our experiment we chose the thickness about 57.9 µm to get better sensing performance.

### 3.3. Detection Process of the Designed IDC Glucose Biosensing System 

**Figure 4:**
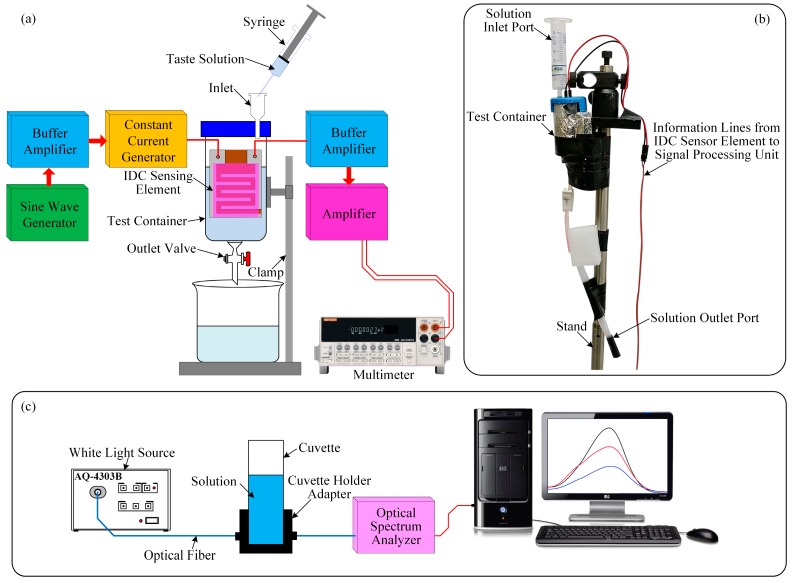
Experimental setup: (**a**) schematic diagram of the IDC glucose biosening system; (**b**) photograph of the various parts of the proposed IDC biosensing system; and (**c**) schematic diagram for measuring the optical properties of different sensing solution under different concentrations of glucose solution.

The experimental setup of the glucose biosensing system is shown in Figure 4a. The setup consists of a test chamber, four interdigitated glucose biosensing elements, a signal processing unit (which consists of an oscillator, two buffer amplifiers, a constant current generator, and an amplifier [37,38,39]), an oscilloscope (TDS3032B, Tektronix, Wilsonville, OR, USA), and a digital multimeter (DMM) (Keithley, 2002, Cleveland, OH, USA). All electronic components of the designed signal processing unit of the developed IDC glucose biosensing system are inexpensive and easily available in the local electronic component market. 

The operation of the proposed IDC glucose biosensor is based on the capacitor principle. Therefore, to produce an electric field between the electrodes of the IDC, we applied an AC voltage of frequency 500 kHz between the terminals of the IDC. The produced electric field enters the dielectric/sensing material, as a result, the impedance of the IDC alter. The change in the concentration of glucose alters the dielectric constant of the sensing material. 

In this experiment, a sine wave oscillator has been designed to generate a frequency of 500 kHz. Its output is connected to the input of a buffer amplifier for reducing the loading effect. The output of the buffer amplifier is connected to the input of a constant current source, and its output is connected to the IDC sensing element. The IDC-sensing-element’s voltage is fed to the input of the buffer amplifier. The output signal of the buffer amplifier is sufficient voltage amplification by an amplifier. The amplifier output is connected to a digital multimeter to measure the response of the IDC biosensing element. 

In our experiment, two types of substances (glucose and sucrose) are individually mixed with deionized (DI) water to get the desired concentrations of the glucose and sucrose solutions from 1 µM to 1 M. The IDC sensing element was placed vertically within the test container. The test container has two ports. To inject the glucose/sucrose solution into the test container, we used a syringe and the inlet port. During this period we closed the outlet valve. After measurement, to remove the test solution or to clean the test container or the IDC sensing element, the outlet valve was left open. 

To measure the sensing response of the different IDC biosensing elements with the glucose and sucrose solutions, first we inject a reference solution by a syringe into the test container to get a stable baseline. Then we slowly inject a target solution into the test container to get a response baseline. When the IDC biosensing element comes into contact with the target solution, then the dielectric constant of the sensing membranes (dielectric material) that was placed into the IDE to form an IDC is changing. As a result, the capacitance of the IDC and the voltage across the IDC also change. The capacitance of the IDC increases with the concentration of the target solution, and *vice versa*. The change in voltage (ΔV) is the difference between the voltage across the IDC for the reference solution and the voltage across the IDC for the target solution. A digital multimeter is used to measure the real-time sensing performance of the IDC sensing element and to record the results. 

The experimental setup for measuring the optical properties of different sensing solution under different concentrations of glucose is shown in Figure 4c, which consist of a white light source of wavelength 400–1800 nm (AQ-4303B, Ando, Kanagawa, Japan), fiber optics, a cuvette, a cuvette holder adapter, an optical spectrum analyzer (QE65000, Ocean Optics, Dunedin, FL, USA), and a computer. Each solvatochromic dye was individually dissolved into the DMAC solution to make 10 mM of different sensing solutions. After that we mixed 0.2 mL of each sensing solution with 0.8 mL of different concentrations of glucose solutions to make different test solutions and observe the absorption peak. We selected the absorption peak of wavelength about 575 nm of the test solutions and were measured by the optical spectrum analyzer which was connected to the computer.

## 4. Results and Discussion

**Figure 5:**
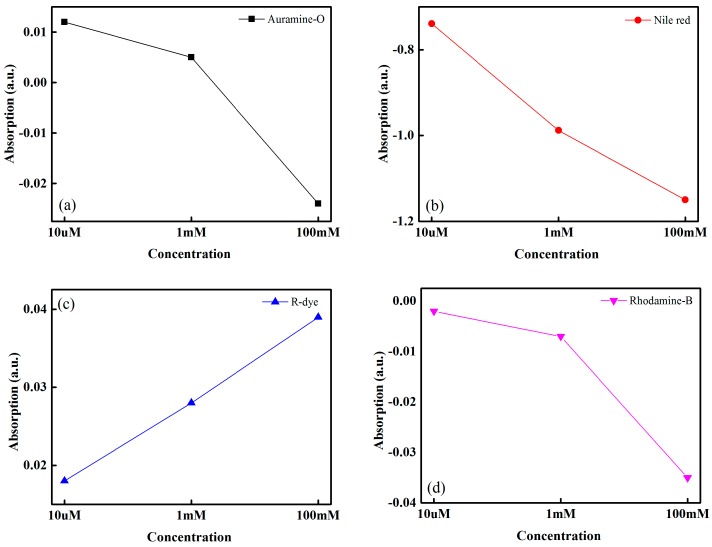
Optical absorption performance of the different dye containing glucose solution: (**a**) Auramine O; (**b**) Nile-red; (**c**) Reichardt’s dye; and (**d**) Rhodamine B.

In our experiment, we used four types of sensing solution to make four different types of IDC glucose biosensing elements. We were able to measure the optical properties of different sensing solutions under different concentrations of glucose. We observed that increasing the concentration of glucose solution in the sensing solution increases the absorption of the sensing solutions. The absorption of the sensing solution changes owing to the charge transfer into the solvatochromic dye as the polarity of the solution changes. From this experiment, it is clear that solvatochromic dye has optical and electrical properties. The optical absorption performance of the various dyes containing glucose solution is shown in Figure 5. R-dye has a negative solvatochromic properties, while the other dyes used in this experiment have positive solvatochromic properties. Therefore, when the solvent polarity of the solution increases due to the increase of the concentration of glucose solution, then the ground state of the R-dye molecule is more polar than the excited state and the specific stabilization occurs in the ground state. As a result, the energy gap between the two electronic states increases, which also increase the transition energy as well as the light absorption peak. Similarly, when the solvent polarity increase then the used positive solvatochromic dye’s excited state goes more polar than the ground state, reduce the transition energy, as well as decrease the absorption peak. Since the relative absorption is the differences from the glucose containing sensing solution (as a test solution) to the DI water containing sensing solution (used as a reference solution). Therefore, in our experiment when the solvent polarity increase, then relative absorption for the R-dye containing sensing solution case increase at the positive direction while in the case of other positive dye containing sensing solutions the absorption is decreasing *i.e.* increasing at the negative direction.

**Figure 6:**
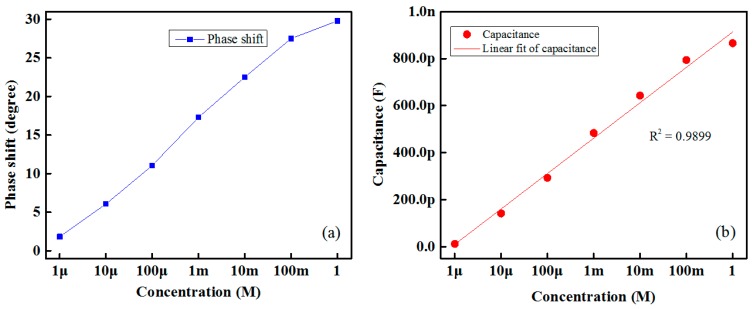
Response of the designed IDC glucose biosensing system: (**a**) change in phase shift of different concentrations of glucose solution; and (**b**) variation in the capacitance.

The proposed IDC glucose biosensing system works on the capacitor principle. Therefore, the phase difference between the signal under reference and the target glucose solution increases as increases the glucose concentration. The result is shown in Figure 6a. The phase differences between the sensing signal and the reference signal for different concentrations of glucose for Nile red containing an IDC sensing element are measured at room temperature using the oscilloscope.

Figure 6b shows the variations in capacitance with respect to different glucose concentrations of the proposed IDC glucose biosensing element, which contained a Nile-red sensing membrane. From this figure it is readily apparent that the change in capacitance of the IDC biosensing element is proportional to the glucose concentration. Its correlation coefficient (R^2^) value is approximately 0.9899.

To observe the sensing response of each biosensing element in glucose and sucrose solutions, we injected glucose and sucrose solutions individually into the test chamber in various concentrations from 1 µM to 1 M. Measurements were taken at room temperature. Since the human taste threshold level of sweetness is about 10 mM (0.01 M) and blood sugar level for diabetes patient before a meal is about 4 to 7 mM/L (4–7 mM) [40,41], in our experiment we took the measurement from 1 µM to 1 M, which covers both sweetness level of food and beverage as well as cover the blood sugar range of diabetes patient. 

**Figure 7:**
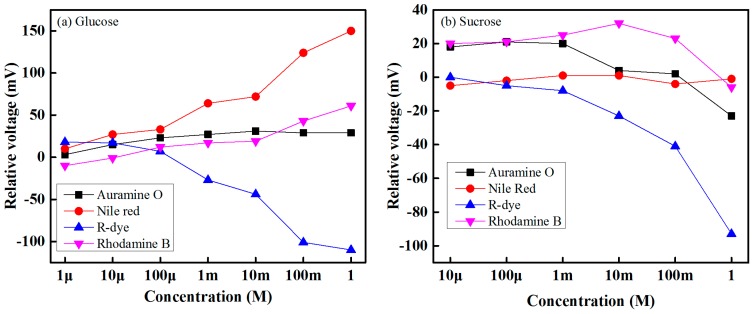
Response of the different sensing elements under: (**a**) glucose solution; and (**b**) sucrose solution.

The sensing performance of the four biosensing elements for glucose with concentrations of 1 µM to 1 M and sucrose solution with concentrations of 10 µM to 1 M are shown in Figure 7. For a given concentration of a target solution, the difference between the obtained voltage of a specific sensing element under the target solution and the reference solution is the response of a sensor. It is seen from Figure 7 that the relative sensing voltage of each sensing element increases as the concentration of the target (glucose or sucrose) solution increases. From these experimental results, it can be determined that each biosensing element that contains different solvatochromic dye gives a sensing response over its dynamic range. In our experiment, we found that the lowest detection rates correspond to a Auramine-O-containing sensing element, while the highest rates correspond to a Nile-red-containing sensing element for the glucose case. On the other hand, for the sucrose case, the lowest detection rates correspond to a Nile-red-containing sensing element, and the highest rates correspond to an R-dye-containing sensing element. 

In our experiment, we used four different kinds of solvatochromic dye containing polymer sensing membranes to make IDC glucose biosensing elements. When the biosensing element is dipped into the glucose solution, then the charge transfer (CT) band of the dye molecule change due to the change of solvent polarity of the glucose solution and the solvent polarity of the glucose solution increase by increasing the concentration of glucose. As a result, the dielectric constant of the sensing membrane as well as the capacitance of the IDC change. Since the charge transfer band of each solvatochromic dye is different; therefore, different IDC biosensing elements offer different sensing abilities at the same concentration of glucose solution.

We fabricated three Nile-red-containing IDC glucose bisensing elements to determine the reproducibility response of the proposed IDC glucose bisensing element. We observed the sensing response of these three IDC glucose biosensing elements and found that all the three show almost the same sensing performance. Therefore, we conclude that IDC glucose sensing elements have excellent reproducibility. The statistical information of the proposed IDC glucose biosensing element at 10 µM of glucose concentration from the three measurements of three specimens of Nile-red-containing sensing membrane is given in Table 1.

**Figure 8:**
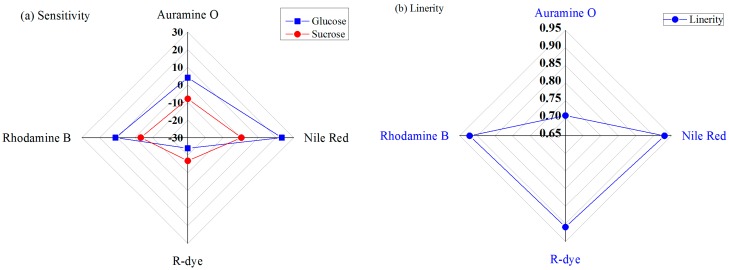
Resopnse of the IDC glucose biosensing system for various dye-containing sensing membranes: (**a**) sensitivity; and (**b**) linearity.

Figure 8a shows the sensitivity performances of the four IDC glucose biosensing elements under different concentrations of glucose or sucrose solutions. It is apparent from this chart that the Nile-red-containing sensing element has a higher sensitivity than the other sensing elements, and that the R-dye-containing sensing element shows a lower sensitivity for glucose. The sensitivities of the designed IDC glucose biosensing element for Nile-red- and R-dye-containing sensing elements for glucose are approximately 23.33 mV/decade and −24.39 mV/decade, respectively.

The linearity response of the developed IDC glucose biosensing system under glucose solutions is shown in Figure 8b. It is found that, the IDC glucose biosensing system has the highest linearity for Nile-red-containing sensing membrane, with an R^2^ value of approximately 0.93088, and the lowest linearity performance for Auramine-O-containing sensing membrane, with an R^2^ value of approximately 0.707.

**Figure 9:**
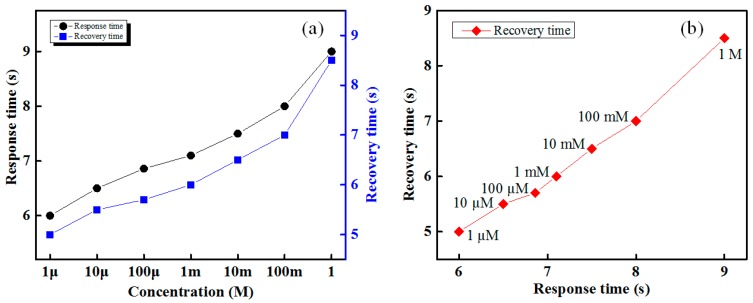
Response of the proposed IDC glucose biosensing system: (**a**) response and recovery times; and (**b**) response *vs.* recovery times at different concentrations of glucose for Nile-red-containing IDC sensing element.

The response and recovery times of the designed IDC glucose biosensing system is shown in Figure 9. The proposed IDC glucose biosensing system has the faster (approximately 7 s) response and a recovery time of 5 s, and those are approximately proportional to the glucose concentration, as shown in Figure 9a. It is apparent from Figure 9b that the response time of various concentration glucose solutions for the Nile-red IDC sensing element is proportional to the recovery time. The developed IDC glucose biosensing system provides a stable sensing response over its dynamic range.

The performance of the proposed IDC glucose biosensing system was tested with different sensing system/principles: IDC [30], potentiometry [42], FET [43], and fiber-optic [44]. These tests were conducted with respect to the response time, dynamic range width, linearity, and sensitivity. We observed that the developed IDC biosensing system provides a wide dynamic range of 1 μM to 1 M (whereas it is 5.5 mM to 28 mM in [30], 0.1 μM to 100 mM in [42], 1 nM to 100 mM in [43], 1 mM to 100 mM in [44]). The response time of the potentiometric [42], FET [43], fiber-optic [44], and electronic tongue SA402 [45] are approximately 6.45 min, <1 s, 10 m, and 20 s, respectively, whereas for the designed IDC glucose biosensing system the response time is less than 7 s. Therefore, it is indicated that the prepared IDC glucose biosensing system offers a fast response time. The sensitivities of the proposed IDC glucose biosensor, potentiometric sensor, and the electronic tongue SA402 [46] for glucose were approximately 23.32 mV/decade, >10 mV/decade, and 40 mV/decade, respectively. From the above result, it can be conclude that the sensitivity of the designed glucose biosensing system was higher than that of the potentiometric sensor and less than that of the electronic tongue SA402. The designed IDC glucose biosensing system gives a linear sensing response over its wide dynamic range and its R^2^ value approximately 0.9308, which is greater than [42]. 

## 5. Conclusions

We proposed an interdigitated capacitor (IDC) glucose biosensing system to measure different concentrations of glucose from 1 μM to 1 M. Four different types of solvatochromic dye were individually incorporated into a polyvinylchloride (PVC) and N,N-Dimethylacetamide (DMAC) solution to make the four dielectric materials used in the four different types of IDC sensing elements. The highly sensitive IDC glucose biosensing system has a short response time of approximately 7 s and a recovery time of 5 s. The dynamic range of the proposed IDC glucose biosensing system varies from 1 μM to 1 M. According to our experimental results, the response property of the sensing system is linear, and its correlation coefficient R^2^ is approximately 0.9308. The reproducibility of the biosensing system is excellent, and its relative standard deviation (RSD) is approximately 0.023. The proposed glucose biosensing system has many advantages including low cost, compactness, and ease of fabrication. Moreover, the designed circuitry can be prepared from easily-obtainable and cheap electronic components. In future studies, we plan to create a taste sensor based on the results of this study. We also plan to make an optical fiber glucose sensing system. 

## Figures and Tables

**Table 1:** Statistical information of the designed IDC glucose biosensing element at 10 µM of glucose concentration for the three measurements of three specimens of Nile-red-containing sensing membrane.

Obs. No.	Relative Voltage (mV)	Standard Deviation of the Relative Voltage
1	26.994	
2	27.029	0.023
3	27.029	
